# A Novel Approach to Addiction Medicine Education for Undergraduate Medical Students

**DOI:** 10.1007/s40670-023-01907-8

**Published:** 2023-10-18

**Authors:** Robert Malinowski, Cara Poland, Jamie K. Alan, Madison O. Walsh

**Affiliations:** 1 Michigan State University, College of Human Medicine, Office of Medical Education Research and Development, East Lansing, MI USA; 2 Michigan State University, College of Human Medicine, Department of Obstetrics, Gynecology and Reproductive Biology, East Lansing, MI USA; 3 Michigan State University, College of Human Medicine, Department of Pharmacology and Toxicology, East Lansing, MI USA

**Keywords:** Addiction medicine, Medical education, Education innovation, Undergraduate medical education, Virtual curriculum, Distance education

## Abstract

A virtual addiction medicine elective was developed using interactive multimedia modules, active learning strategies, and patient-based cases. Student had opportunities for professional networking and interacting with physicians and patients. The elective was successful in boosting Year-1 medical students’ confidence to screen, manage, and treat patients with substance abuse disorder.

## Innovation Manuscript

The 2020 National Survey on Drug Use and Health reported 40.3 million (14.5%) Americans aged 12 or older were living with a substance use disorder (SUD) in the past year [1]. According to the CDC’s National Vital Statistics System (NVSS), drug overdose deaths from November 2020 through October 2021 included a record high of 105,752, a 15.9% increase from the prior year [2]. There is a critical need of more education for healthcare providers to meet the needs of the population. Prevention, management, and treatment of people with substance use disorders (SUDs) is often neglected in undergraduate medical education, and few medical colleges have an appropriate number of curricular hours devoted to addiction medicine, when compared to other chronic diseases [3]. In this paper, we describe a robust active-learning centered addiction medicine elective, utilizing biomedical, clinical, and social science aspects, aligned to increase students’ knowledge, awareness, and skills in identifying, assessing, and treating persons with SUDs.

In May of 2021, during the COVID-19 pandemic, the virtual 4-week addiction medicine elective was launched. Dynamic and interactive media-rich modules were developed to deliver pre-learning materials. The course utilized active learning strategies, including exercises based on problem-based learning (PBL) and team-based learning (TBL) techniques. Content was designed to be integrated and applicable to realistic clinical situations. Students participated in interactive large group experiences, along with focused small group discussions. The discussions utilized PBL principles, where the students engaged with an interactive patient-based case that addressed basic, social, and clinical science principles. Board-style questions were incorporated into sessions using TBL principles, which included an individual readiness assessment test (iRAT) and team readiness assessment test (tRAT). The weekly wrap-up session reviewed content using Kahoot, an interactive, game-like software. Students were assessed by the IRAT/tRAT tests and by completing a reflective statement. Content experts, as well as patients, participated in question-and-answer sessions to further explore topics and give their own unique perspectives. Beyond didactics, the course offered beneficial intangibles including opportunities for professional networking, casual chatting with physicians, and career planning advice in preparation for practicing in the field of addiction medicine. Thirty-six, Year-1 medical students completed a pre-post self-reported survey, adapted from the previously validated substance abuse attitudes Likert scale survey (SAAS), to assess attitudes and the impact of these benefits. Because contemporary evidence continues to show poor patient outcomes resulting from unconscious bias related to stigma, the field continues to monitor attitudes using the SASS survey. We used this survey with minor modifications, which were done to eliminate stigmatizing language. The survey included statements regarding permissiveness, treatment intervention, treatment optimism, and non-moralism. Students were surveyed pre/post, and again at 3 months and 6 months.

Table 1 reports the survey attitudes towards SUD treatment. When asked if SUD is a treatable disease (Question 6, Table 1), the majority of pre-course attitudes were reported as “agree” (51%) compared to the majority of post-course survey attitudes, were reported as strongly agree (60%). Another key question to compare attitudes is Question 9, Table 1, “A person with substance use disorder is unpleasant to work with as a patient”. Approximately 68% either disagreed or strongly disagreed in the pre-survey, compared to an increase to 77% in the post-survey. Students are aware of social discrimination and strive to improve outcomes for underserved patients. They recognize the need for addiction education and, when given the opportunity, take electives in addiction medicine. Students with early exposure have improved attitudes toward persons with addiction that sustain for at least six months beyond the course. Training programs should educate medical students about SUDs in the early stages of their careers, rather than expecting students to seek further education after graduation. Consistent early training and education can identify, enable, and equip students to serve as champions and future leaders in addiction medicine.

This elective course used a variety of teaching models to keep students engaged, present integrated content, and enable learners to apply it to realistic clinical situations. Equally important were the opportunities for networking, career development and interacting with physicians. Our results show that an elective addiction medicine curriculum can boost confidence in the ability to screen, manage, and treat patients with SUD. This was a pilot study, and we recognize our small sample size is a limitation. While the focus of this elective was addiction medicine, the same techniques could be applied to other disciplines. Schools should consider exposing students to topics often neglected in mainstream medical curricula, where stigma and a lack of education often cause social, medical, and treatment inequities.

**Table 1:**
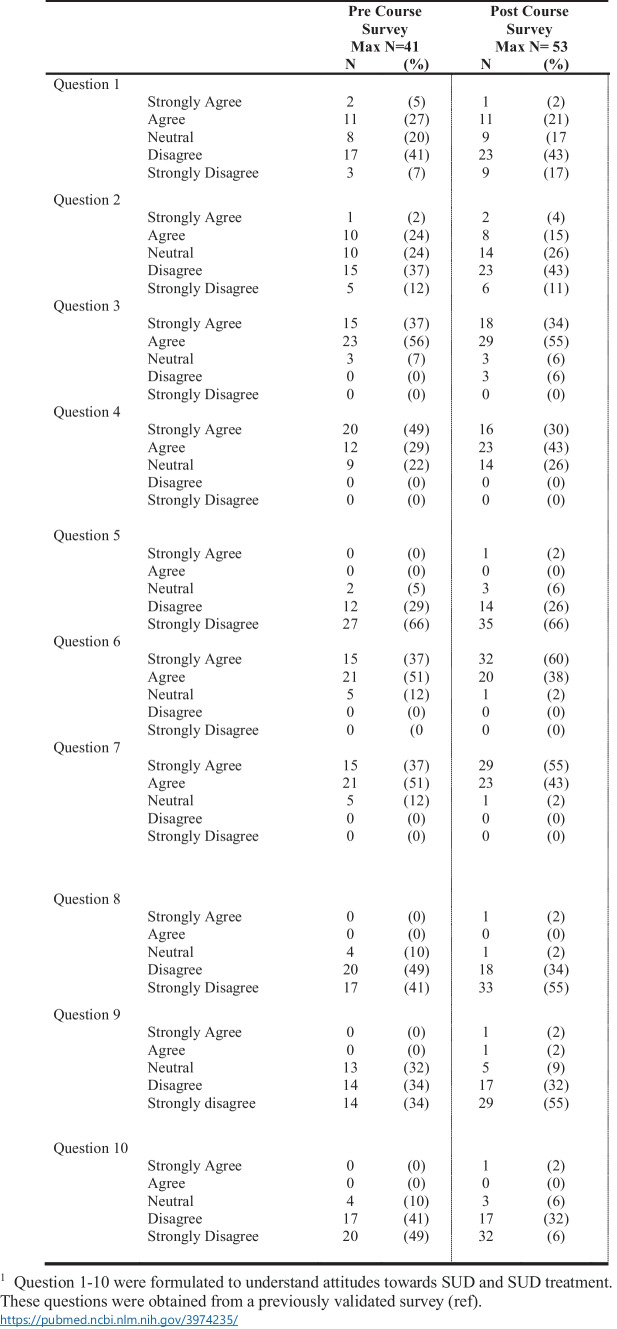
Pre and Post Course Survey Attitudes 2021

## Data Availability

The data used was anonymized and accessed through the Michigan State University College of Human Medicine.
